# Exosomes as Messengers between Mother and Fetus in Pregnancy

**DOI:** 10.3390/ijms21124264

**Published:** 2020-06-15

**Authors:** Liliana Czernek, Markus Düchler

**Affiliations:** Department of Bioorganic Chemistry, Centre of Molecular and Macromolecular Studies, Polish Academy of Sciences, 112, Sienkiewicza Street, 90-363 Lodz, Poland; lilianaczernek@gmail.com

**Keywords:** placenta, exosomes, extracellular vesicles, pregnancy, embryo implantation

## Abstract

The ability of exosomes to transport different molecular cargoes and their ability to influence various physiological factors is already well known. An exciting area of research explores the functions of exosomes in healthy and pathological pregnancies. Placenta-derived exosomes were identified in the maternal circulation during pregnancy and their contribution in the crosstalk between mother and fetus are now starting to become defined. In this review, we will try to summarize actual knowledge about this topic and to answer the question of how important exosomes are for a healthy pregnancy.

## 1. Introduction

Intensive maternal-fetal information exchange is required to establish and to maintain a healthy pregnancy. The placenta is the most important organ in this respect, performing vital functions for the fetus to support its growth and survival and to maintain the pregnancy [1]. The placental assignments also include the control and regulation of the communication between the mother and the developing child [2]. In general, cellular communication is mediated through direct cell-to-cell contacts, soluble factors, intercellular nanotubes, and extracellular vesicles [3]. Placental cells of both maternal and embryonic origin, secrete not only soluble endocrine mediators but also extracellular vesicles, including exosomes. For the maternal immune system, a pregnancy poses an exceptional challenge as the embryo constitutes a foreign tissue in immunological terms that must not be destroyed. A finely tuned immunosuppression has to take place to avoid rejection of the embryo. In addition, other pregnancy complications can result from dysfunctional placental communication causing severe danger for the survival of the fetus [4]. In this review, we summarize the role of exosomes in healthy and pathological pregnancies.

## 2. Exosomes and Other Extracellular Vesicles

**Figure 1:**
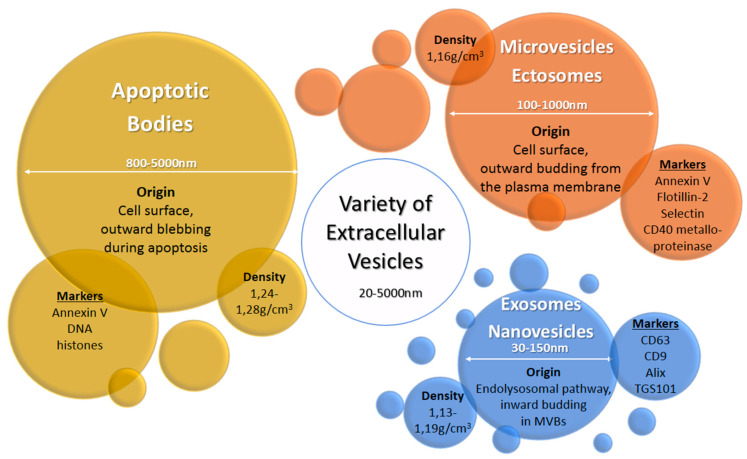
Origin, size, density, and typical markers of major extracellular vesicle subclasses [6,7].

Extracellular vesicles (EV) are heterogeneous populations of cell-derived membrane vesicles released by eukaryotic and prokaryotic cells to the extracellular space. The classification of EVs is based on the origin and size of the vesicle (Figure 1) [5]. The human placenta releases a wide variety of EVs including macrovesicles (syncytial nuclear aggregates, 20–100 μm), microvesicles, apoptotic bodies, and nanovesicles (exosomes) [3,6]. The function of syncytial nuclear aggregates is unclear as they can contain tens or hundreds of nuclei and could represent the remnants of dying syncytiotrophoblasts. Microvesicles are budding from the cell membrane and typically show a diameter of 100 to 1000 nm. Exosomes were considered originally to be cellular “debris”, but do in fact play an important role in the body as mediators of intercellular communication. They are the smallest representatives of EVs with a diameter of 30 to 150 nm and are produced by a defined pathway. Budding from the membrane of the multivesicular body, a part of the endosomal compartment, their composition including their cargo loosely follows certain formation rules resulting in a heterogeneous population of vesicles—with some communalities. Although the origin of microvesicles and exosomes is well known, the experimental discrimination of these vesicles types is difficult, so the terms are sometimes subsumed as extracellular vesicles [6]. In this review, we use the terms exosomes or extracellular vesicles according to the usage in the reviewed publications. Exosomes consist of a lipid bilayer with the same orientation as the plasma membrane and contain a variety of proteins and nucleic acids—some of which are enriched especially in these vesicles. Their content varies due to cell type and environment conditions. They are produced by almost every cell type—as well by cancer cells. Once released into the extracellular space, exosomes may act locally to modify the activity of neighboring cells or distally after entry into circulating bodily fluids. Exosomes were found in most biological fluids including blood, lymph, saliva, milk, amniotic fluid, lachrymal and mammary gland secretions [7].

## 3. The Human Pregnancy

After fertilization, the zygote starts to move through the fallopian tube where the first mitoses occur [8]. The first cell doublings result in the morula, comprising 16 cells, which develops further into the blastocyst. The blastocyst reaches the uterus and implants into the modified part of the endometrium of the uterus called decidua. The blastocyst contains two kinds of cells that are the product of the first differentiation processes, the inner cell mass, and the surrounding trophoblasts. The inner cell mass develops into embryoblasts that form the embryonic disc. In addition, the cells of the trophoblast undergo further differentiation into an inner layer comprising the cytotrophoblasts (CT; sometimes villous cytotrophoblasts, VCT), and an outer layer containing the syncytiotrophoblasts (SCT). In the blastocyst stage, the embryonic anlage is nurtured by diffusion of nutrients from the maternal blood. The placenta is a temporary organ that develops when the blastocyst becomes implanted into the maternal endometrium and is composed of cells from both the embryo and the uterus. Villous tree structures are formed that contain a network of blood vessels. In the placenta, an intensive exchange of oxygen, CO_2_, and nutrients takes place between the maternal and the embryonic circulation. The embryonic disc undergoes further differentiation processes that give rise to the gastrula, a trilaminar disc comprised of the three germ layers ectoderm, mesoderm, and endoderm. During the next weeks, early progenitors for all kinds of tissues are formed. The growing embryo detaches from the uterus wall staying connected through the umbilical cord. The cord contains blood vessels of the embryonic circulation and connects it to the placenta where material transfer takes place without direct mixing of the embryonic and maternal blood.

## 4. The Placenta as the Interface Between Maternal and Fetal Organisms

**Figure 2:**
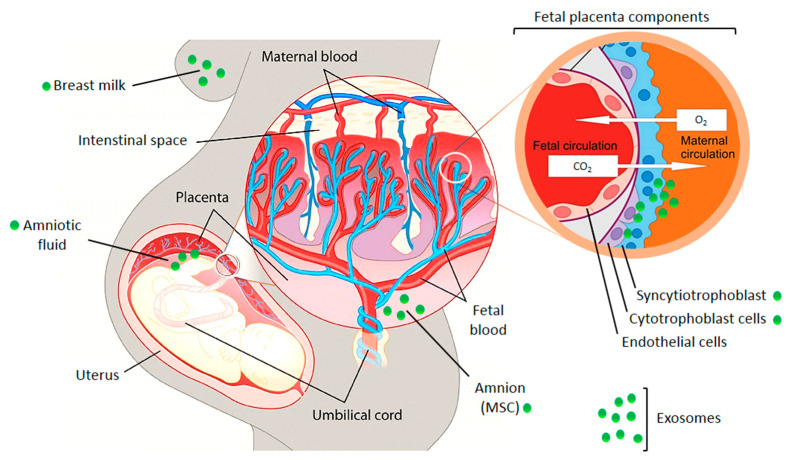
Illustration of the fetal placental barrier that separates fetal and maternal circulations in the human placenta. Places of exosome appearance are indicated. MSC—mesenchymal stem cells. The picture was composed using publicly available graphics from The Alcohol Pharmacology Education Partnership at the Duke University Medical Center [13] and from Christiane Albrecht, University of Bern, with her friendly permission [14].

The crosstalk between fetus and mother can occur as a simple diffusion of molecules through tissue layers or in a better protected manner through extracellular vesicles, especially via exosomes (Figure 2) [9,10]. Embryo-derived exosomes encapsulate a variety of different proteins and nucleic acids (microRNA, messenger RNA, long non-coding RNA, DNA) and can subsequently be taken up by cells of the maternal immune and vascular systems. They modulate the maternal physiology to cause or adapt it to pregnancy-induced changes [9]. The release of placenta derived exosomes into the maternal circulation has been demonstrated in healthy and pathologic pregnancies [3,9,10]. Placenta-derived exosomes may be differentiated from other exosomes by the presence of placenta-specific miRNAs or proteins. One of them is placental alkaline phosphatase (PLAP), a syncytiotrophoblast-specific protein. Moreover, trophoblast-derived exosomes carry characteristic trans-membrane proteins such as human leukocyte antigen G (HLA-G) [11]. A recent study demonstrated that the total amount and the specific placenta-derived exosomes could be determined using quantum dots coupled with CD63 and PLAP antibodies, respectively. Quantification of placental exosomes in maternal plasma reflects fetal growth and it may be a useful indicator of placental function [12].

Fetal-derived exosomes travel to the maternal side to potentially transmit signals to the uterus and cervix. It was even suggested that inflammatory signals delivered by exosomes could contribute to the onset of parturition [15]. Exosomes as potential paracrine mediators triggering labor were also identified in mice. Birth could be experimentally induced by exosomes in the absence of systemic progesterone withdrawal, which is otherwise a requirement for birth initiation [9].

Through the effects on the maternal organism, the released exosomes also support growth and survival of the fetus, therefore one would expect an increased production of exosomes during pregnancy. This hypothesis was confirmed, as the concentration of exosomes in maternal peripheral blood was ~20-fold greater than that observed in non-pregnant women [16]. The concentration of placenta-derived exosomes in maternal plasma increases in normal pregnant women as gestation progresses, with a maximum concentration reported at term [3,17]. The increase with gestational age can be observed already during the first trimester of pregnancy, starting as early as in the sixth week—even before the intervillous circulation is fully developed. Hypoxia and/or hyperglycemia inside the uterus result in an increased exosome release from syncytiotrophoblasts which can facilitate extravillous trophoblast invasion and proliferation [18].

In general, research techniques to study the placenta-derived exosomes use cell cultures of trophoblasts (primary and cell lines), chorionic villi explants, placental perfusion, plasma and urine from pregnant women. Main areas of interest about exosomes in pregnancy include: demonstration of the exchange of EVs between fetus and mother; the role of EVs in implantation; the establishment of immune tolerance; and the regulation of angiogenesis and endothelial cell migration.

### 4.1. EVs Are Exchanged Between Maternal and Embryonic Tissues

The human placenta releases a wide range of molecules and EVs which support the maternal physiology to adapt to fetal requirements during pregnancy [10,19,20]. Placenta-derived EVs (including microvesicles and exosomes) from normal human pregnancies in the first-trimester are absorbed by endothelial cells through phagocytosis and endocytosis [21]. In a mouse model, placental vesicles targeted specific organs in vivo, particularly the lungs, the liver, and the kidneys. These findings were confirmed by employing fetal cell-derived fluorescently labelled exosomes that were injected intra-amniotically into pregnant mice [9]. Exosomal trafficking and function was further demonstrated by using genetically engineered mice in which fetal and maternal exosomes could be distinguished [22]. Both feto-maternal and maternal−fetal trafficking of exosomes during pregnancy was demonstrated by this elegant approach, as exosomes from the mother were able to cross placental barriers and reach fetal tissues. The detection of fetal exosomes in maternal plasma discloses their potential as biomarkers for pregnancy monitoring using minimally invasive liquid biopsy.

### 4.2. Exosomes in Pregnancy Transfer miRNAs to Regulate Gene Expression in Target Cells

One class of small RNAs transported via extracellular vesicles, the microRNAs (miRNAs), attracted special attention. MiRNAs are post-transcriptional regulators of gene expression by inhibiting the translation of their target mRNAs or inducing mRNA degradation. It was estimated that the expression of 60% of the human genes are regulated by miRNAs [23]. The loading of miRNAs into exosomes occurs through controlled pathways [24]. MiRNAs are encoded by the genome and their transcription underlies cell program dependent regulation similar to protein coding genes. The analysis of the miRNA content in exosomes gives information about the status of the producer cell, a fact that makes them useful for diagnostic purposes. Taken up by recipient cells, the miRNAs transported by exosomes influence translation and can profoundly change the gene expression pattern. Experimental loading of exosomes with specific miRNAs is intensively explored for the transport of therapeutic nucleic acids in future therapies.

Characteristic miRNAs, which are highly expressed in human placentas, were identified in the serum of pregnant women. For instance, the concentration of placental miRNA-141 increases in maternal plasma with gestational age [25]. Several miRNAs are located in the chromosome 19 miRNA cluster (C19MC) which is the largest cluster of miRNAs in the human genome [26]. MicroRNAs within the human C19MC include 46 miRNAs which are expressed only in the placenta, the so-called placenta-associated miRNAs [27,28]. During pregnancy, C19MC-derived miRNAs are expressed in villous trophoblasts and secreted into the maternal circulation via exosomes where they function in placental-maternal signaling [29,30]. For instance, exosomal miR-517b increased the expression of TNFα and/or other death ligands [30]. The exosomal transfer of placenta-specific miR-571a-3p into NK cells repressed cGMP-dependent protein kinase 1, a key mediator of nitric oxide signaling [31]. In pregnant mice, 15 miRNAs specific for pregnant animals were identified in trophoblast-derived EVs [32]. Among the potential target pathways identified by bioinformatics analysis was the ubiquitin-mediated proteolysis, MAPK signaling and Focal Adhesion Pathways.

Interestingly, there is evidence that exosomes can help the fetus to avoid viral infections. Non-placental cells incubated with placental exosomes became more resistant to viral infection and this effect was mediated by the delivery of specific miRNAs [33]. Recently, another class of small RNAs have been identified in syncytiotrophoblast-derived EVs, fragments of transfer RNA (tRNA) [34]. tRNA can be split by specific enzymes into halves that exert various intracellular functions in stress signaling [35]. Transported by vesicles, tRNA halves were shown to interfere with protein synthesis in target cells [34].

### 4.3. Exosomes Support the Implantation of the Embryo

A metadata analysis of the function of extracellular vesicles in the human reproduction system suggested that they contribute to oocyte and sperm maturation, fertilization, prevention of polyspermy, and embryo implantation [36]. During the window of implantation, the embryonic blastocyst adheres to the uterus wall and initiates cell invasion. Exosomes help to establish the correct coordination between the embryo and uterine endometrium required for successful implantation [36,37]. MiRNAs have been detected as mediators of embryo-endometrium crosstalk in this process [38,39]. Exosomes released from the endometrial epithelium into the uterine cavity transfer specific miRNAs to the trophectodermal cells of the blastocyst or to endometrial epithelial cells to promote implantation [40]. Comparing the miRNAs of the exosomes and their producer cells demonstrated controlled sorting of miRNA into exosomes, and 13 of the 227 miRNA identified were specific for exosomes. Investigation of the potential pathways that are regulated by these specific exosomal miRNAs may lead to the identification of targets in pathways promoting embryo implantation [40,41]. By bioinformatics analyses, potential target genes related to ECM-receptor interactions, the Jak-Stat pathway, VEGF signaling, and Toll-like receptor signaling were identified.

Exosomes derived from human endometrial epithelial cells were shown to be taken up by trophoblasts to enhance their adhesive potential partially by enhanced focal adhesion kinase signaling [42]. In addition, in a mouse model, miRNAs present in exosomes were shown to be transferred from the receptive endometrial epithelium to embryonic trophectoderm, improving the adhesive ability of the pre-implantation embryo. Exosome-derived miR-30d could induce overexpression of genes involved in embryo adhesion, such as integrin beta-3, integrin alpha-7, and cadherin-5 [43]. A regulatory function for cell invasion was described for exosomal miR-520c-3p from chorionic villous trophoblasts. MiR-520c-3p targeted CD44 in extravillous trophoblasts, and downregulation of endogenous miR-520c-3p accelerated EVT invasion [44].

Evaluating exosomes in the uterine luminal fluid of sheep demonstrated that vesicle transported proteins could affect the implantation and fertility outcomes. Released by the endometrial epithelium, vesicles were taken up by blastocysts and the endometrium around the uterus [45]. A total of 195 vesicular proteins were identified by nano-LC-MS/MS analysis with 40 and 76 unique to the cyclic and pregnant ewes, respectively. Mass spectrometry analysis of the isolated extracellular vesicles found many proteins expressed by the endometrial epithelia and/or conceptus trophectoderm including cathepsin L1, gastrin releasing peptide, lipoprotein lipase, and prostaglandin synthase 2 [45].

### 4.4. The Influence of Pregnancy-Associated EVs on the Maternal Immune System

The fetus is antigenically distinct from the mother and therefore it is necessary to establish a tolerant immune state to prevent the rejection of the developing organism [46]. The role of exosomes released by embryonic cells and the placenta seems to be crucial for the fetus to evade the maternal immunosurveillance and destruction of the fetal ‘allograft’. The immunosuppressive potential of exosomes is well recognized in cancer [47]. Cancer-derived exosomes prevent the differentiation and activation of immune effector cells, modulate antigen expression, induce T cell apoptosis, and transport immunosuppressive cytokines. Recently, we provided evidence that melanoma-derived EVs were able to confer antigen-specific immunosuppression by simultaneously transporting MHC molecules presenting cancer-specific antigenic peptides and immunosuppressive cytokines [48]. Antigen-specific immunosuppression seems to occur during pregnancy as well and could be partially mediated by EVs. Embryo-derived exosomes are taken up by cells of the maternal immune system, resulting in inhibition of the host immune system to facilitate the establishment of pregnancy, fetal development, and survival during pregnancy [49]. Exosomes derived from villous cytotrophoblasts (VCT) reduced the production of Th1 cytokines in PBMCs. Exosome-associated syncytin-2 (Syn-2), an immunosuppressive molecule, mediated this effect, as it was not observed in Syn-2-silenced VCT exosomes [50]. Most importantly, exosomes can inhibit activation of T-lymphocytes and natural killer cells that are potentially harmful to the embryo [51]. The immunosuppressive character of placenta-derived vesicles is partially mediated by the expression of death messengers including the pro-apoptotic molecules FasL (Fas ligand), PD-L1 and TRAIL [16]. Engagement of the cognate receptors triggers apoptosis in activated peripheral blood mononuclear cells (PBMCs) [52,53]. In a normal pregnancy, placental exosomes become critical in modulating T-cell activation, suppressing effector T cells by enhancing lymphocyte apoptosis and CD3-zeta loss [16]. CD3 loss affects the selection of T lymphocytes leading to decreased T lymphocyte-mediated responses. Embryonic exosomes also down-regulate the activating NK cell receptor NKG2D during pregnancy impairing NKG2D-mediated cytotoxicity [52,53]. NKG2D is down-regulated on NK cells by exposure to its soluble ligands [54]. The NKG2D ligands MIC-A and MIC-B are expressed by the placenta and are released via exosomes to inhibit NK cell activity.

Trophoblast-derived EVs were further shown to induce the differentiation of T cells into Treg (regulatory T) cells [55]. Treg cells exert a strong antigen-specific immunosuppression [47]. The Treg induction was mediated by HSPE1 (heat shock 10kDa protein 1) carried by the EVs [55].

In cattle, placenta-derived exosomes are abundant in the circulation of pregnant cows and exosome-derived miR-499 attenuated the expression of proinflammatory cytokines by inhibiting NF-κB signaling, thereby attenuating inflammatory responses and forming an immune-tolerant microenvironment in the uterus. Inhibition of miR-499 lead to inflammatory deregulation and increased risk of pregnancy failure [56].

A role in preventing the embryo from being attacked by the maternal immune system was described for glycosphingolipids (GSLs) in exosomes [57]. GSL expressing exosomes derived from villus trophoblast cells significantly induced macrophage M2 polarization during a normal pregnancy. Mature macrophages can acquire a pro-inflammatory M1 phenotype or become polarized towards an immunosuppressive M2 state [47].

Inflammation is part of an active immune response. Considering the necessity for inducing tolerance for the embryo, is might seem counterintuitive that the implantation requires a slightly inflammatory status. However, this condition does not affect the fetus directly, but facilitates tissue remodeling and embryo implantation [58]. Placental vesicles are thought to play a role in the maternal systemic inflammatory response by modulating cytokine release [59]. Exosomes isolated from pregnant women promoted the release of proinflammatory cytokines (IL-4, IL-6, IL-8, IFN-γ, TNF-α) from human umbilical vein endothelial cells (HUVECs) and this effect was significantly greater when exosomes isolated from gestational diabetes mellitus (GDM) pregnancies were used [59]. In addition, macrophage-derived exosomes internalized by placental cells increased the release of pro-inflammatory cytokines such as IL-6, IL-8 and IL-10 [60].

### 4.5. The Angiogenic Potential of Exosomes in Pregnancy

Initial stages of pregnancy are characterized by spiral artery remodeling and physiological adaptations in the cardiovascular system to provide sufficient supply of nutrients and oxygen to the growing fetus [61]. Coordinated fetal vasculogenesis and maternal vascular remodeling requires invasion and differentiation of trophoblast cells. The invasive cytotrophoblasts replace the endothelial layer of the maternal spiral arteries, transforming them from high-resistance vessels into large-scale capacitance vessels suitable for sufficiently nourishing the fetus [62]. Placenta-derived EVs are reported to induce vasculogenesis and angiogenesis through an oxygen-sensing mechanism, especially under the hypoxic conditions during the early stages of pregnancy. Furthermore, vascular endothelial growth factor A (VEGFA), an angiogenesis stimulator and vessel growth factor, along with exosomal miRNAs, is released by the implanted embryo to regulate blood supply [63]. Both, maternal and umbilical serum exosomes enhance endothelial cell proliferation, migration, and tube formation. In umbilical serum exosomes, altered expression of a subset of migration-related miRNAs including miR-210-3p, miR-376c-3p, miR-151a-5p, miR-296-5p, miR-122-5p, and miR-550a-5p has been identified [64].

Exosomes derived from porcine trophectoderm cells induced the proliferation of the maternal endothelial cells and promoted angiogenic processes due to the presence of specific miRNAs. Increased miR-150 levels in the EVs stimulated the proliferation and migration of endothelial cells, thereby exhibiting a pro-angiogenic effect. A reduced expression of porcine miR-150 in the umbilical cord blood-derived exosomes of pigs showed intrauterine growth restriction [65]. MiRNAs involved in modulation of angiogenesis at the maternal-fetal interface were identified in porcine trophectoderm cell lines. 14 miRNAs were selectively present in CD63 positive EVs of which miR-126-5P, miR-296-5P, miR-16, and miR-17-5P were the most abundant angiogenic miRNAs [66].

## 5. Exosomes in Pathological Pregnancies

Placental exosomes are reported to be involved in the pathology of pregnancy as well [3,67]. The quantity and content of placental exosomes could be linked to placental dysfunction—specifically to preeclampsia, gestational diabetes, and preterm birth [3,19,68]. Increased secretion of vesicles was detected during pregnancies complicated by gestational diabetes [20] and preeclampsia [69]. Partially, differences in exosomal miRNA levels could be associated with these pathological states. Besides, cell-free DNA (cfDNA) of exosomal origin is considered as biomarker of pregnancy complications [70,71].

### 5.1. Preeclampsia

One of the most serious and common complications of pregnancy is preeclampsia (PE). It occurs especially in the third trimester of pregnancy. PE and associated hypertensive disorders are responsible for nearly 40% of premature births [72,73]. In the pathogenesis of PE, placental exosomes show variations in quantity and protein cargo, and exert some impact on maternal immune tolerance. In mouse models, EVs derived from injured placentas were shown to induce PE including hypertension and proteinuria [74]. The number of extracellular vesicles in pregnant women was tested as predictive marker for PE. The total number of microvesicles was significantly elevated in plasma obtained from women with normal pregnancy and late-onset PE compared with non-pregnant women matched by age, but there was no significant difference in pathological and normal pregnancies [75]. Shedding of vesicles and debris from trophoblast cells into the maternal circulation during PE contributed to vascular inflammation and endothelial injury, which are associated with the pathophysiology of PE [76]. Placental syncytiotrophoblast-derived extracellular vesicles contain endothelial nitric oxide synthase (eNOS), an enzyme that produces nitric oxide (NO) required for the regulation of the vascular tone and blood supply [77]. Based on diminished NO biological activity, EVs enable the prediction of pregnant disorders including PE [78]. Other studies also related low NO with placenta-derived exosomes. The exosomal miR-155, which is highly expressed in the plasma and placenta of preeclampsia patients, inhibited the expression of eNOS in endothelial cells [79]. Relevant for hypertension, EVs released by placental syncytiotrophoblasts carry neprilysin, an enzyme from the family of membrane-bound metalloproteases. Neprilysin cleaves vasopeptides, thus contributing to the establishment of hypertension, a hallmark of PE [80]. Interestingly, the rate of early onset of PE in high risk women could be significantly reduced by aspirin and this effect was mediated by down-regulation of miR-155 transported by EVs [81,82].

Both, exosomal miRNAs and proteins, were successfully employed as predictive biomarkers [83]. Small RNA sequencing of serum derived samples from women who later developed PE allowed for the discovery of miRNAs whose levels were deregulated in this condition [84]. The identified miRNAs were compared to the miRNAs found by 19 preceding studies pursuing similar aims. An overlap was found for 11 up-regulated and 5 down-regulated miRNAs in PE as compared to healthy pregnancies. MiR-155 was again among the up-regulated miRNAs. Furthermore, three miRNAs (miR-26b-5p, miR-7-5p, and miR-181a-5p) previously associated with hypertension, one of the hallmarks of PE, were among those deregulated in PE [84]. In another study, hsa-miR-486-1-5p and hsa-miR-486-2-5p were identified as microRNAs with predictive potential [85].

Proteomic studies identified about 400 proteins in syncytiotrophoblast EV samples with 25 proteins (including integrins, annexins, and histones) unique to PE compared to healthy pregnant controls [86]. Syncytin-2 was less abundant on the surface of serum-derived exosomes isolated from patients with PE. The levels of specific proteins in exosomes in pregnant women, such as syncytin-2, can be used as biomarkers for the prediction and diagnosis of PE [50,87]. Isolation of sub-populations of exosomes using their affinity to Cholera toxin B chain or annexin V followed by protein quantification by ELISA allowed to identify PE-predictive biomarkers. Three proteins, Tissue Inhibitor of Metalloproteinases 1 (TIMP1), Plasminogen Activator Inhibitor Type I (PAI1), and Placental Growth Factor (PlGF), showed excellent predictive robustness [88]. Furthermore, a combinatorial measurement of copeptin, Placental Growth Factor and Annexin V-positive microparticles could be used for PE prediction and discrimination from other pregnancy complications [89]

### 5.2. Pre-Tterm Birth

According to the World Health Organization, an estimated 15 million infants are born too early each year [90]. Complications following preterm birth are the leading cause of death among children under five years of age and are responsible for about one million deaths each year globally. A pre-term birth in humans is defined as birth before 37 completed weeks of gestation. PE is one of the major medical causes of pre-term birth.

In pregnant mice, the concentration of exosomes was significantly lower in preterm birth induced by inflammation compared to animals with normal delivery [91]. Pathway analysis after determination of exosomal protein profiles at term and preterm birth pointed at changes in inflammatory and endocrine signaling, which might disrupt pregnancy maintenance [92]. To test the influence of exosome signaling on parturition timing, vesicles were isolated from the blood of pregnant mice either during early or late pregnancy. Then, these EVs were injected into a separate group of pregnant mice in a pregnancy stage corresponding to the beginning of the third trimester in human [93]. The injection of a high concentration of late pregnancy exosomes was able to cause labor-associated changes without the other hormonal and chemical triggers usually involved in this process. In contrary, the injections of the early pregnancy exosomes had no effect [93].

A range of miRNAs isolated from maternal plasma were described as predictive biomarkers for pre-term birth, however, whether these miRNAs were transported by EVs was not evaluated in these studies [94,95,96,97]. Identification of miRNAs transported by EVs also revealed potential biomarkers for the prediction of pre-term birth [98]. Changes of the protein composition of circulating placental EVs could also be used for the identification of a high-risk status for pre-term birth across gestation [99]. Ninety-six proteins were expressed at significantly different levels, and a bioinformatics analysis revealed their connection to inflammatory pathways, epithelial mesenchymal transition, and coagulation/complement activation.

### 5.3. Gestational Diabetes Mellitus (GDM)

Another important complication of pregnancy, which can harm both the fetus and the mother, is gestational diabetes mellitus (GDM). During normal pregnancy, at the time of the second and third trimester, limited insulin resistance gradually increases as a normal phenomenon to ensure sufficient nutrient supply for the fetus. A pregnant mother with pathologic insulin resistance—acquired or chronic—is not able to compensate for the increased circulating glucose concentrations because of β-cell dysfunction. As a result, maternal hyperglycemia occurs, leading to increased risk of disease for both the mother and the fetus [100]. GDM is defined by any degree of abnormal glucose metabolism diagnosed during pregnancy and/or glucose intolerance that was not present or recognized before pregnancy. GDM associated hyperglycemia, hyperinsulinemia, and hypoxia may adversely affect the maternal-fetal vascular exchange and placentation, leading to preterm birth, fetal distress, fetal death, and other adverse pregnancy outcomes. GDM was associated with elevated levels of exosomes in the maternal circulation. Placental exosomes from GDM pregnancies decreased insulin-stimulated migration and glucose uptake in primary skeletal muscle cells obtained from patients with normal insulin sensitivity. In contrary, the exosomes derived from normal glucose tolerant patients increased the glucose uptake in response to insulin in skeletal muscle cells from diabetic subjects [101]. A connection between increased exosome levels and glucose concentration in GDM was also described in first-trimester trophoblast cells inducing the release of cytokines from endothelial cells [102]. It was suggested that increased placental glycogenolysis in GDM accelerates glucose transfer to the fetus, resulting in fetal overgrowth. There is a significant association between high body weight and diabetes, and adipose tissue-derived exosomes were shown to influence the placental glycogenolysis. Pathway analysis of exosomal proteins revealed differential expression of mitochondrial function-related proteins in adipose tissue-derived exosomes of GDM [103].

A proteomic study identified 78 proteins with significantly altered levels in GDM-derived exosomes compared to normal ones. Pathway analysis revealed that many of these proteins are involved in energy production and inflammation [104]. Analysis of urinary exosomes revealed that the damage associated molecular pattern (DAMP) protein S100A9 was present at higher levels in exosomes in GDM and could be used as valid biomarker of inflammatory processes and immune responses [105]. In addition, increased levels of miRNA members of the C19MC region were found in exosomes during GDM: miR-518a-5p, miR-518b, miR-518c, miR-518e, miR-520c-3p, and miR-525-5p [106,107]. Furthermore, the expression of specific exosomal miRs including miR-125a-3p, miR-99b-5p, miR-197-3p, miR-22-3p, and miR-224-5p were detected at elevated levels in the placenta, in circulating exosomes and in skeletal muscle in GDM [101]. Several studies analyzed the miRNA changes in GDM in plasma samples without isolating EVs [108,109,110]. All of them identified candidates suitable for GDM prediction. The usage of exosomes to treat GDM was suggested recently [111].

## 6. Conclusions

**Figure 3:**
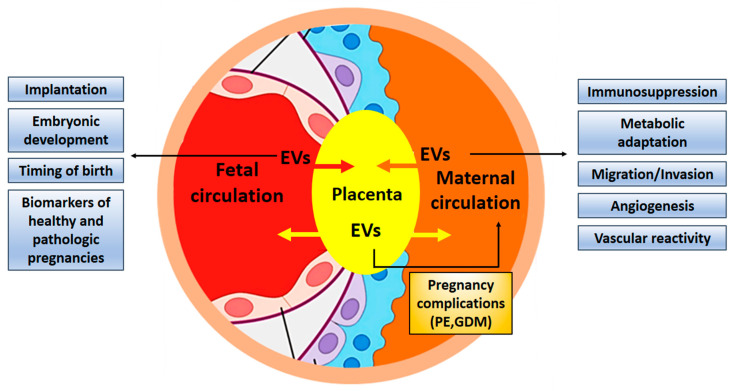
Exosomes contribute to fetal-maternal communication. Exosomes are exchanged between maternal and embryonic tissues to facilitate implantation and development of the embryo, adapting the maternal organism for pregnancy by suppressing immune responses and guaranteeing nutritional supply. Exosomes can also contribute to pregnancy complications and might be used as biomarkers. EV, extracellular vesicles; PE, preeclampsia; GDM, gestational diabetes mellitus.

The contribution of exosomes in fetal-maternal communication during pregnancy has been firmly established (Figure 3). It is even conceivable that exosomes are necessary for the successful implantation of the embryo and its normal development. Despite growing interest in elucidating the role of exosomes during normal and complicated pregnancies, progress in the field seems to be quite slow. One of the reasons may be connected to problems with the isolation of placental exosomes from the maternal circulation. An improved workflow for their isolation was recently described [112,113]. Changes in exosomal concentration, composition, and/or their bioactivity, such as interaction with maternal cells, may participate in the development of pathological states, and exosomes can be used as biomarkers in the prediction of pregnancy complications such as preeclampsia, fetal growth restriction, and preterm birth [29,114,115,116]. Furthermore, exosomes have the potential to serve as new therapeutic targets in infertility [117]. The unique characteristics of exosomes and their ability to carry cargo to distant destinations in the body makes them ideal candidates to signal between feto-maternal tissues during pregnancy [15]. Studying the contribution of exosomes in pathological pregnancies and related diseases, such as infertility or pregnancy failure can open the way for new exosome-based therapies [118].

## Abbreviations

EV	extracellular vesicle
CT	cytotrophoblast
VCT	villous cytotrophoblasts
SCT	syncytiotrophoblast
PLAP	placental alkaline phosphatase
HLA-G	human leukocyte antigen G
MSC	mesenchymal stem cells
C19MC	chromosome 19 miRNA cluster
TNFα	tumor necrosis factor α
ECM	extra-cellular matrix
VEGF	vascular endothelial growth factor
EVT	extravillous trophoblast
LC-MS	liquid chromatography–mass spectrometry
FasL	Fas ligand
PD-L1	programmed death ligand-1
TRAIL	TNF- Related Apoptosis Inducing Ligand
PBMCs	peripheral blood mononuclear cells
HSPE1	heat shock 10kDa protein 1
GSL	glycosphingolipid
IL-4	interleukin-4
IFN-γ	interferon-γ
HUVEC	human umbilical vein endothelial cell
cfDNA	cell-free DNA
eNOS	endothelial nitric oxide synthase
NO	nitric oxide
PE	preeclampsia
GDM	gestational diabetes mellitus
DAMP	damage associated molecular pattern
